# Polyploid Genome Assembly Provides Insights into Morphological Development and Ascorbic Acid Accumulation of *Sauropus androgynus*

**DOI:** 10.3390/ijms25010300

**Published:** 2023-12-25

**Authors:** Fagang Xia, Bin Li, Kangkang Song, Yankun Wang, Zhuangwei Hou, Haozhen Li, Xiaohua Zhang, Fangping Li, Long Yang

**Affiliations:** 1 Key Laboratory of Genetics, Breeding and Multiple Utilization of Crops, Ministry of Education, College of Agriculture, Fujian Agriculture and Forestry University, Fuzhou 350002, China; sdxfm@163.com (F.X.); yankunwang@fafu.edu.cn (Y.W.); 2 Key Laboratory of Biological Breeding for Fujian and Taiwan Crops, Ministry of Agriculture and Rural Affairs, College of Agriculture, Fujian Agriculture and Forestry University, Fuzhou 350002, China; 3 Agricultural Big-Data Research Center, College of Plant Protection, Shandong Agricultural University, Tai’an 271018, China; libin2994429664@163.com (B.L.); kangkangsong234@163.com (K.S.); 2022120120@sdau.edu.cn (H.L.); 15052036269@163.com (X.Z.); 4 Guangdong Provincial Key Laboratory of Plant Molecular Breeding, State Key Laboratory for Conservation and Utilization of Subtropical Agro-Bioresources, South China Agricultural University, Guangzhou 510642, China; alfredhou8@gmail.com

**Keywords:** *Sauropus androgynus*, genome assembly, Phyllanthaceae, polyploidy, herbal medicine, ascorbic acid

## Abstract

*Sauropus androgynus* (*S. androgynus*) (2*n* = 4*x* = 52) is one of the most popular functional leafy vegetables in South and Southeast Asia. With its rich nutritional and pharmaceutical values, it has traditionally had widespread use for dietary and herbal purposes. Here, the genome of *S. androgynus* was sequenced and assembled, revealing a genome size of 1.55 Gb with 26 pseudo-chromosomes. Phylogenetic analysis traced back the divergence of *Sauropus* from *Phyllanthus* to approximately 29.67 million years ago (Mya). Genome analysis revealed that *S. androgynus* polyploidized around 20.51 Mya and shared a γ event about 132.95 Mya. Gene function analysis suggested that the expansion of pathways related to phloem development, lignin biosynthesis, and photosynthesis tended to result in the morphological differences among species within the Phyllanthaceae family, characterized by varying ploidy levels. The high accumulation of ascorbic acid in *S. androgynus* was attributed to the high expression of genes associated with the L-galactose pathway and recycling pathway. Moreover, the expanded gene families of *S. androgynus* exhibited multiple biochemical pathways associated with its comprehensive pharmacological activity, geographic adaptation and distinctive pleasurable flavor. Altogether, our findings represent a crucial genomic asset for *S. androgynus*, casting light on the intricate ploidy within the Phyllanthaceae family.

## 1. Introduction

**Figure 1:**
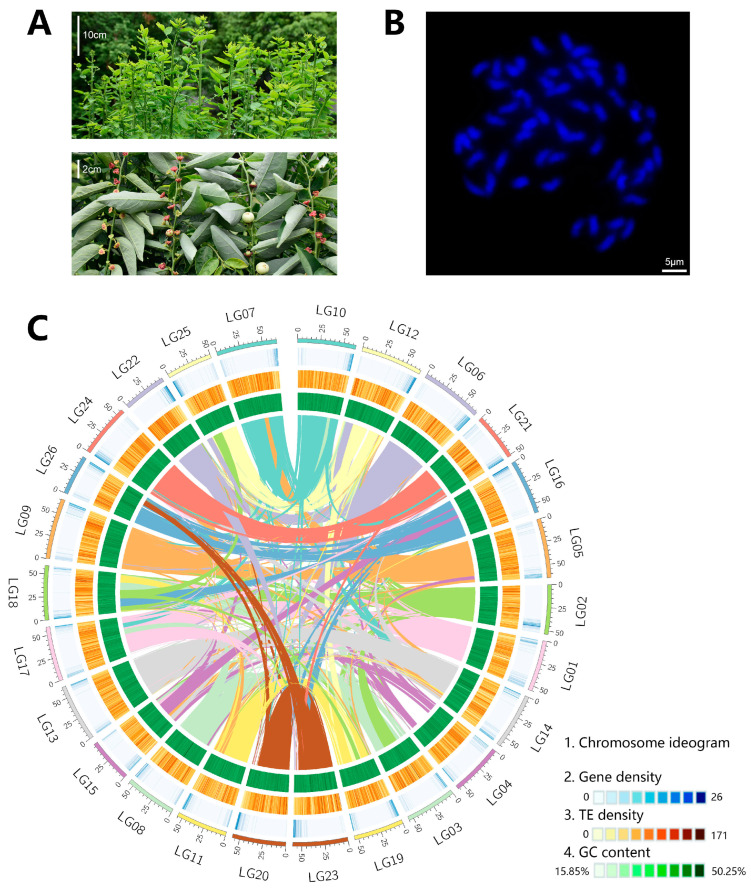
The basic morphology of *S. androgynus* and basic characteristics of its genomes. (**A**) Morphological characteristics of *S. androgynus*. The top picture provides an overview of the whole bush; the bottom picture highlights the morphological characteristics of the plant, including its leaves, flowers and fruits. (**B**) Image of the karyotype in *S. androgynus*. (**C**) Landscape of the *S. androgynus* genome: (1) Chromosome ideogram, (2) Gene density, (3) TE density, and (4) GC content. The inner circle represents the collinear blocks identified in its genome.

*Sauropus androgynus* L. Merr (*S. androgynus*) (2*n* = 4*x* = 52; family: Phyllanthaceae; Figure 1A) is a perennial shrub that naturally thrives in hot and humid environments, exhibiting wide dispersion and cultivation across South and Southeast Asia [1]. The plant has been traditionally used as an herbal remedy for various ailments, including colds, eye diseases, and gastrointestinal disorders [2,3,4]. Additionally, it holds high culinary value in countries like India, Indonesia, and Malaysia [5,6].

As one of the most popular functional foliar vegetables in South and Southeast Asia, the popularity of *S. androgynus* is attributed not only to the exceptionally delightful flavor of its leaves [7] but also to its wealth of nutritional and pharmaceutical values. *S. androgynus* is abundant in a variety of body-healthy components, with numerous publications reporting its abundance of ascorbic acid (vitamin C), vitamin A, vitamin E, flavonoid, phenol, etc. [2,8,9,10,11]. An earlier study showed that the content of vitamin C in fresh leaves of *S. androgynus* was 314.3 mg/100 g, which was higher than in common edible vegetables [2]. As a multivitamin plant, the high content of vitamin C in *S. androgynus* has also been demonstrated in many other studies [1,6,8]. These bioactive components present in *S. androgynus* can serve as direct or potential sources of its antioxidant, antibacterial and anti-inflammatory abilities [5,12,13], which provide the fundamental basis for its comprehensive pharmacological activities.

The current research on *S. androgynus* mainly concentrates on its phytochemical and biological components, as well as its pharmacology applications. However, the absence of reference genome sequences and limited functional genomics studies have hindered the comprehension of their underlying molecular mechanisms, thus presenting challenges for advancing related research.

The genus *Sauropus* has been confirmed to be deeply embedded in the Phyllanthaceae by molecular approaches (such as ITS and matK) [14]. In an early classification, the Phyllanthaceae family was categorized as the subfamily Phyllanthoideae, under the Euphorbiaceae family [15]. However, the revised APG II classification recognized Phyllanthaceae as a distinct and independent family within the order Malpighiales [16]. Despite this, probably due to the different taxonomic principles, or the speed of updating the website, certain websites (e.g., Integrated Taxonomic Information System (https://www.itis.gov/ (accessed on 30 March 2023)), PLANTS Database (https://plants.usda.gov/ (accessed on 30 March 2023)), Plants of the World Online (https://powo.science.kew.org/ (accessed on 30 March 2023)), The International Plant Names Index (https://www.ipni.org/ (accessed on 30 March 2023)), iPlant (http://www.iplant.cn/ (accessed on 30 March 2023))) and recent studies [17,18] still classify the taxonomy of *S. androgynus* under the Euphorbiaceae family. As such, we hope to furnish further genetic evidence to clarify the evolutionary placement of the Phyllanthaceae family.

Polyploidization events often exhibit a correlation with enhanced vigor and the adaptation of newly formed polyploids to novel environmental conditions, which has been widely utilized in the breeding processes of crops [19]. These evolutionary events may induce morphological changes in species, such as the enlargement of plant organs following polyploidization [20]. Previous studies involving karyotype analysis have identified diverse chromosomal counts in plant specimens belonging to this botanical family [21]. This observation implies the presence of distinct ploidy levels among species within the family. The first genome assembly of diploid Phyllanthaceae (*Phyllanthus cochinchinensis*), with a genome size of 284.88 Mb and 13 pseudo-chromosomes, has been released based on the combination of Illumina, PacBio, and Hi-C technology [22]. This has promoted further functional genomic research on species within the Phyllanthaceae family. However, only a diploid genome is clearly insufficient for comprehensive research on a family characterized by complex ploidy.

In this study, we employed various methods to determine the ploidy of *S. androgynus* and achieved a chromosomal-level assembly of its genome. Our subsequent exploration via functional gene analysis unveiled specific genes affected by polyploidy, which likely play a role in driving morphological variations. Furthermore, we conducted an extensive investigation into the highly scrutinized vitamin C metabolic pathway in *S. androgynus*. The outcomes of this research are anticipated to provide valuable insights for future genetic studies within the Phyllanthaceae family, while also holding significance for the medicinal applications of *S. androgynus*.

## 2. Results

### 2.1. Potential Polyploid of S. androgynus

Previous research had reported a diploid species, *P. cochinchinensis*, with 13 pairs of homologous chromosomes (2*n* = 26; *n* = 13) [21]. Karyotype analysis in *S. androgynus* detected a twofold (2*n* = 52) elevation in the chromosomes number in the pachytene of the root tips cells (Figure 1B). The estimation of the genome size based on k-mer analysis and flow cytometer elucidated the elevation in the genome size (1.43 Gb and 1.52 Gb) in *S. androgynus* (Figures S1 and S2). Smudgeplot analysis revealed the characteristic of the allotetraploids in *S. androgynus* (Figure S1). These results suggested the potential polyploid in *S. androgynus*.

### 2.2. Genome Assembly and Quality Assessment

A total of 44.32 Gb (~31.03×) PacBio circular consensus sequencing (CCS) and 192.75 Gb (~134.79×) Hi-C Illumina platform clean data were combined to assemble the *S. androgynus* genome sequence. Initially, we assembled the reads into 1.68 Gb of contig sequences, with a contig N50 size of 29.83 Mb (Table S1). The scaffold, based on Hi-C data, generated a genome assembly with a size of 1.55 Gb and a scaffold N50 of 58.10 Mb (Table S1). A total of 1.52 Gb (97.79%) of assembled sequences were anchored onto 26 pseudo-chromosomes, with 1.50 Gb (96.28%) of sequences successfully establishing their order and orientation. The results corresponded to the chromosome numbers in the karyotype assay and elucidated a chromosome-level genome assembly for *S. androgynus* (Figure 1C and Figure S3, Table S2).

The quality and coverage of the genome assembly were evaluated from the perspectives of completeness in the gene and genome sequence. Benchmarking Universal Single-Copy Orthologs (BUSCO) and Core Eukaryotic Genes Mapping Approach (CEGMA) analyses predicted 96.84% complete BUSCO genes and 98.47% CEGMA genes in the *S. androgynus* genome assembly, respectively (Tables S3 and S4). Additionally, 96.92% of the short-read sequences were properly mapped to the assembly (Table S5). These assessments collectively suggested a high-quality genome assembly for *S. androgynus*.

### 2.3. LTR Accumulation Promoted the Genome Expansion of S. androgynus

In the genome of *S. androgynus*, 77.81% of the total length was classified as repetitive sequences using both homology-based and de novo methods (Table 1). Among these sequences, transposable elements constituted 74.02% of the genome length (Table S6). The predominant type of repeat was retroelements, accounting for 71.25% of the genome length. Further analysis of these repetitive sequences revealed that the long terminal repeat (LTR) superfamily elements, including LTR/Copia, LTR/Gypsy, and LTR/Unknown, constituted 36.27%, 20.3%, and 13.65% of the genome length, respectively. LTR insertion time analysis showed a gradual accumulation of these LTRs over 5 million years, peaking at 0.13 million years ago (Mya) (Figure S4). Additionally, the genome of *S. androgynus* contained 3.79% tandem repeats (Table S6).

### 2.4. Gene Prediction and Annotation of S. androgynus

Through the integration of homology-based, de novo, and RNA-Seq data-based prediction methods (Table S7 and Figure S5), a total of 26,048 protein-coding genes were identified within the *S. androgynus* genome. The average lengths of the whole genes, coding sequences, exon sequences, and intron sequences were found to be 3405.95, 1354.96, 1739.48, and 1666.47 base pairs (bp), respectively (Table S8). BUSCO assessment revealed 96.47% completeness, indicating the high quality of the gene prediction (Table S9). A total of 99.36% of the protein-coding genes were functionally annotated using various databases: Gene Ontology (GO) (84.6%), Kyoto Encyclopedia of Genes and Genomes (KEGG) (77.53%), KOG (58.2%), Pfam (88.57%), SwissProt (85.18%), TrEMBL (99.3%), EggNOG (87.52%), and NR (99.17%) (Table S10 and Figure S6). Additionally, apart from the protein-coding genes, the analysis also revealed a range of non-coding RNAs: 8705 rRNAs, 5422 tRNAs, 152 microRNAs, 48 snRNAs, and 72 snoRNAs (Table S11). Alongside this, 149 pseudogenes were cataloged.

### 2.5. Evolution History and Comparative Analysis of S. androgynus

**Figure 2:**
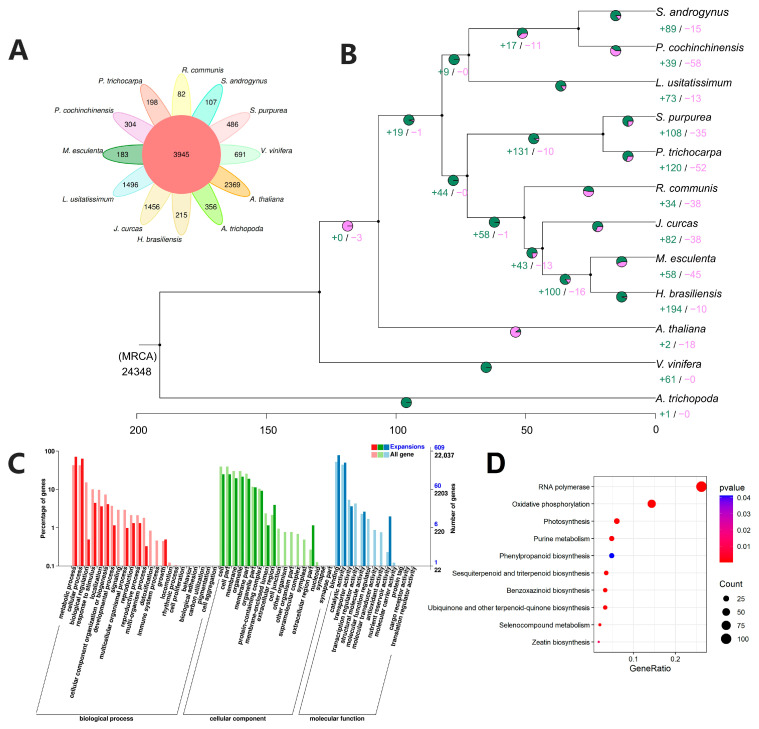
Evolutionary history and comparative analysis of *S. androgynus*. (**A**) Venn diagram of the species-specific and shared gene families across *S. androgynus* and 11 other species (*A. thaliana*, *A. trichopoda*, *H. brasiliensis*, *J. curcas*, *L. usitatissimum*, *M. esculenta*, *P. cochinchinensis*, *P. trichocarpa*, *R. communis*, *S. purpurea* and *V. vinifera*). (**B**) Phylogenetic relationship of the 12 species. The divergence times are labeled at the bottom. The numbers on each branch represent the expansion (green) and contraction (red) of gene families. (**C**) GO and (**D**) KEGG enrichment of the expanded genes in *S. androgynus*.

To investigate the genome evolution and divergence of *S. androgynus*, phylogenomic analysis were conducted using protein sequences from *S. androgynus* and 11 angiosperm species, including one Phyllanthaceae plant (*P. cochinchinensis*), four Euphorbiaceae plants (*Hevea brasiliensis*, *Jatropha curcas*, *Manihot esculenta* and *Ricinus communis*), two Salicaceae plants (*Sarracenia purpurea* and *Populus trichocarpa*), one Linaceae plant (*Linum usitatissimum*) and three model plants (*Arabidopsis thaliana*, *Amborella trichopoda* and *Vitis vinifera*) (Table S12). All the protein-coding genes were clustered into 32,293 orthogroups based on the sequence homology. Additionally, we identified a total of 3945 gene families shared among all 12 species, along with 107 species-specific gene families in *S. androgynus* (Figure 2A and Table S13). KEGG enrichment analysis of these genes revealed enrichment in various pathways, including RNA polymerase, biotin metabolism, fatty acid biosynthesis and metabolism, purine metabolism, 2-oxocarboxylic acid metabolism, protein export, ascorbate and aldarate metabolism (Figure S7).

The phylogenetic tree was developed based on 213 single-copy orthologs. Phylogenetic analysis indicated that *S. androgynus* and *P. cochinchinensis* diverged about 29.67 Mya (Figure 2B and Figure S8). Phyllanthaceae and Euphorbiaceae shared a common ancestor about 82.3 Mya. The formation of these two families was associated with the divergence of Linaceae and Salicaceae.

The dynamic of gene family analysis detected 89 and 15 gene family expansions and contractions in the *S. androgynus* genome (Figure 2B). GO enrichment analysis indicated the genes in the expansion family related to the metabolic process, cellular process, response to stimulus, growth, catalytic activity, transporter activity, nutrient reservoir activity, and so forth (Figure 2C). These findings suggested the occurrence of frequent biochemical reactions internally, which corresponded to the biosynthesis and metabolic pathways of multiple compounds, as revealed by KEGG enrichment analysis (Figure 2D).

### 2.6. Polyploidization and Synteny Analysis of S. androgynus

A whole-genome duplication (WGD) event is an important force in plant evolution [23,24]. Previous analysis in this research indicated the potential polyploid in *S. androgynus*. Multiple apparently self-syntenic segments were observed within the *S. androgynus* genome (Figure S9). Synteny analysis between *S. androgynus* and a diploid near species, *P. cochinchinensis,* revealed numerous collinear blocks and an apparent 2:1 projection ratio between the two genomes. This suggested a tetraploid (2*n* = 4*x* = 52) in *S. androgynus*.

**Figure 3:**
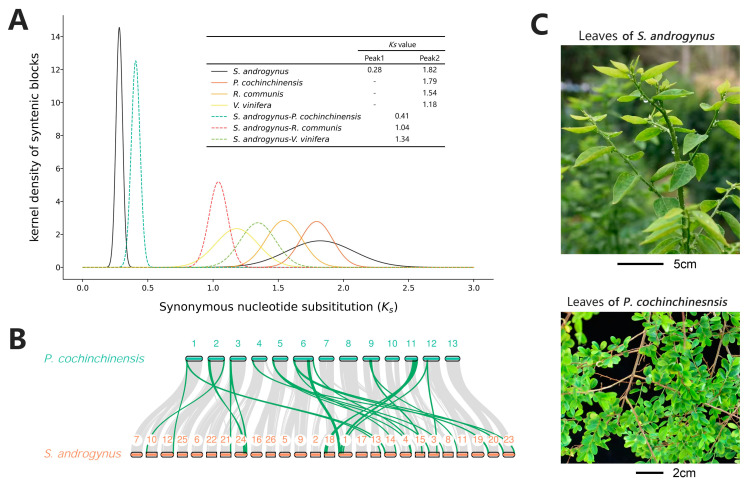
Whole-genome duplication analysis, synteny analysis, and morphological comparison of *S. androgynus* and *P. cochinchinensis*. (**A**) Ks distribution of *S. androgynus*, *P. cochinchinensis*, *R. communis* and *V. vinifera*. (**B**) Synteny analysis between *S. androgynus* and *P. cochinchinensis*. The green blocks display the potential chromosomal translocation events. The number indicates the chromosome number for each genome. (**C**) Morphological comparison of a leaf for *S. androgynus* (**up**) and *P. cochinchinensis* (**below**).

The distributions of the synonymous substitutions per synonymous site (*Ks*) values of *S. androgynus* indicated a recent WGD event about 20.51 Mya. This time was later than the divergence between *S. androgynus* and *P. cochinchinensis,* which suggested that the ancestors of these two species were diploid (Figure 3A). It is noteworthy that, from the perspective of tetraploidy, the duplicate rate in the *S. androgynus* genome is relatively low (7.31%; Table S3). This indicated a substantial loss of redundant genes within this genome, reflecting an ongoing diploidization process. In addition, the *S. androgynus* and *P. cochinchinensis* genomes shared an ancient WGD event, which was the common whole-genome triplication (γ event) shared by all the core eudicots (Figure 3A) [25].

### 2.7. Expansion of Genes Related to Morphological Development

**Figure 4:**
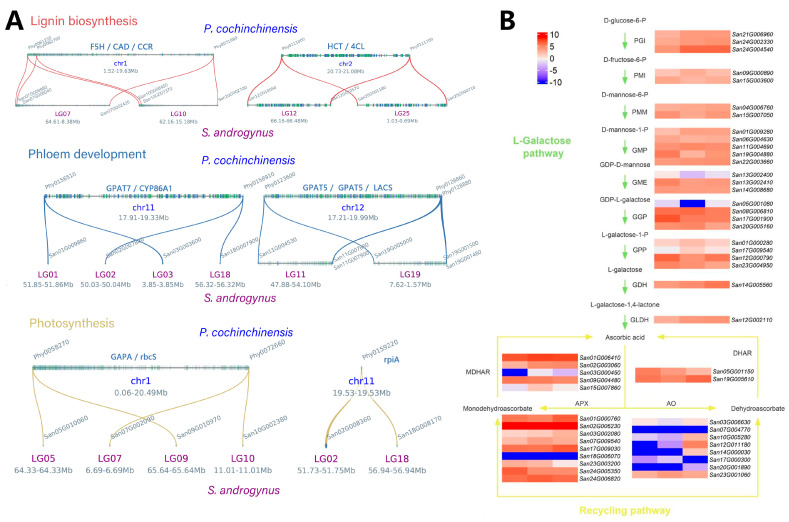
Microsynteny of genes related to morphological development, and gene expression patterns in the ascorbic acid metabolic pathway of *S. androgynus*. (**A**) Microsynteny of genes related to morphological development in *S. androgynus* and *P. cochinchinensis*. From the top to the bottom are the microsynteny patterns of lignin synthesis, phloem development, and photosynthesis. (**B**) Gene expression patterns in the ascorbic acid metabolic pathway of *S. androgynus*. The heatmaps in each row correspond to one gene, which refers to a leaf, stem and flower from left to right, respectively, and its expression level is quantified by log2 (FPKM + 0.001). Abbreviations: PGI: glucose-6-phosphate isomerase, PMI: mannose-6-phosphate isomerase, PMM: phosphomannomutase, GMP: GDP-D-mannose pyrophosphorylase, GME: GDP-D-mannose-3,5-epimerase, GGP: GDP-L-galactose phosphorylase, GPP: L-galactose-1-phosphate phosphatase, GDH: L-galactose dehydrogenase, GLDH: L-galactono-1,4-lactone dehydrogenase, APX: L-ascorbate peroxidase, AO: L-ascorbate oxidase, MDHAR: monodehydroascorbate reductase, DHAR: dehydroascorbate reductase.

Morphological differences are common among related species with different ploidy [26,27]. Tetraploid *S. androgynus* exhibits more robust stems and a larger area of mature leaves than diploid *P. cochinchinensis* (Figure 1A and Figure 3C) [22]. Botanical studies have proven that vascular development and lignin biosynthesis are closely related to these morphological characteristics [28,29]. Functional analysis illustrated the expansion of multiple genes encoding the key enzymes of lignin biosynthesis in tetraploid *S. androgynus*, including *PAL*, *HCT*, *CCR* and *CAD* (Figure 4A and Figure S10). A similar pattern was detected in genes associated with phloem development (*FAR4/5*, *KCS2/20*, *LACS*, *GPAT5/7* and *CYP86A/B1*). Downstream, genes related to photosynthesis also exhibited similar characteristics, which tended to provide sufficient metabolic material for the development of the larger morphological features of *S. androgynus*.

### 2.8. Transcriptional Regulation of Ascorbic Acid Accumulation in S. androgynus

The high concentration of ascorbic acid, a key component of the antioxidant system, is a distinguishing feature of *S. androgynus*. To investigate the expression levels of genes associated with ascorbic acid accumulation, transcriptome data from three distinct tissues of *S. androgynus* were analyzed.

Functional annotation revealed a total of 52 genes associated with the biosynthesis and recycling pathways of ascorbic acid in *S. androgynus* (Table S13). Within the biosynthesis pathway specific to ascorbic acid, all the genes associated with the L-galactose pathway were identified, whereas deletions were observed in the remaining pathways, including those involving GalUR (D-galacturonate reductase in the galacturonate pathway), Alase (aldonolactonase in the galacturonate pathway), and GLOase (L-gulonolactone oxidase in both the myo-inositol and L-gulose pathways) (Figure S11 and Table S13).

The gene expression patterns within the L-galactose pathway were found to be consistent across the leaf, stem, and flower tissues, and the elevated expression of related genes corresponded to the high vitamin C content of *S. androgynus* (Figure 4B). Furthermore, all the genes associated with the ascorbic acid recycling pathway were identified. A comparison revealed that while the expression of AO-related genes was low, there was pronounced expression of APX- and MDHAR-related genes (specifically *San02G006230* and *San01G006410*). This pattern indicates that the recycling pathway of ascorbic acid in *S. androgynus* primarily relies on the conversion of monodehydroascorbate into ascorbic acid.

## 3. Discussion

*S. androgynus*, which is rich in a variety of nutrients and biomolecules, is not only an important edible vegetable in some countries and regions but is also used in traditional remedies for its comprehensive pharmacological activities. In this study, we assembled a high-quality *S. androgynus* genome based on a combination of Illumina, PacBio and Hi-C technology. The genome, with a total length of 1.55 Gb, consists of 26 pseudo-chromosomes and comprises 26,048 predicted protein-coding genes. The chromosome-level genome assembly and annotation of *S. androgynus* provides new insights into the intricate ploidy within the Phyllanthaceae family and facilitates further medicinal applications of *S. androgynus*.

Phylogenetic analysis indicated that *S. androgynus* and *P. cochinchinensis* diverged about 29.67 Mya (Figure 2B). In addition, Phyllanthaceae and Euphorbiaceae shared a common ancestor around 82.3 Mya. The formation of these two families was accompanied by the divergence of Linaceae and Salicaceae. These results provide deeper genomic insights into the taxonomic relationship between the Phyllanthaceae and Euphorbiaceae families, as well as the differentiation of various families within the order of Malpighiales.

Polyploidy plays an important role in evolution, and it is an important mechanism for species formation and adaptation to environmental variations [30,31,32]. The *Ks* distribution suggested that *S. androgynus* experienced a γ event about 132.95 Mya and a species-specific WGD event about 20.51 Mya (Figure 3A). The synteny patterns within the *S. androgynus* genome (Figure 1C) and between the genomes of *S. androgynus* and *P. cochinchinensis* (Figure 3B) suggested two highly similar yet distinct subgenomes within *S. androgynus*. Furthermore, it is noteworthy that *S. androgynus* exhibits a relatively low duplication rate (7.31%; Table S3), indicating a substantial loss of redundant genes in its genome, which reflects an ongoing diploidization process.

In general, polyploidy tends to enlarge cell and organ sizes, and it can even influence the overall growth habit of organisms [33]. Additionally, morphological differences are commonly observed among species with varying ploidy levels [26,27]. In comparison to *P. cochinchinensis*, *S. androgynus* exhibits more robust stems and larger mature leaf areas (Figure 1A and Figure 3C). Microsynteny analysis reveals the expansion of genes related to morphological development within *S. androgynus*, including lignin biosynthesis, phloem development, and photosynthesis (Figure 4A and Figure S10), which provide an ample supply of metabolites to support the development of the larger morphological features of *S. androgynus*. Furthermore, it is noteworthy that most of these expanded genes in *S. androgynus* are distributed among groups of homologous chromosomes, which tend to originate from polyploidy events. These results suggest that the number of genes amplified by polyploidy contributed to the plant magnification in the *S. androgynus* tetraploid.

As a traditional herb widely used in certain South and Southeast Asian countries, *S. androgynus* exhibits comprehensive pharmacological activities, including antioxidant, antibacterial and anti-inflammatory activities. GO enrichment of the annotated genes in the genome of *S. androgynus* demonstrated the enrichment of antioxidant activity, detoxification, immune system process, and so forth (Figure S6), which was in correspondence with its comprehensive pharmacological activities. In terms of the molecular mechanism, these effects may result from the expansion of the biosynthesis or metabolic pathways of multiple compounds with antioxidant capacity, such as phenylpropanoid, sesquiterpenoid, triterpenoid, benzoxazinoid and selenocompounds (Figure 2D). This suggests that the comprehensive pharmacological activity of *S. androgynus* could stem from cumulative effects and interactions of multiple genes.

The ability of ascorbic acid to supply electrons makes it a free radical scavenger [34], which is related to the antioxidant activity in plants. In addition to its excellent antioxidant capacity, previous studies have shown that ascorbic acid can act as an essential micronutrient in the human body due to its anti-aging [35] and even anti-cancer [36] effects. The absence of the L-gulono-γ-lactone oxidase (GLO) gene disrupts the synthesis of ascorbic acid [37], making the food intake the main source of ascorbic acid in humans. Consequently, the genetic basis underlying the accumulation and genetic improvement of ascorbic acid, primarily obtained from plants [38], has been extensively studied [39,40,41,42]. Similar to many plants, *S. androgynus*, which adopted the L-galactose pathway as the primary biosynthetic pathway, exhibited high expression of related genes and a high content of ascorbic acid [41,43]. Although several gene copies were identified in AO, the low expression level of related genes indicates that the recycling pathway of ascorbic acid in *S. androgynus* does not primarily depend on the transformation of the intermediate product dehydroascorbate to ascorbic acid, as catalyzed by AO (Figure 4B). In contrast, the high expression of APX- and MDHAR-related genes in the ascorbic acid recycling pathway promotes the transformation of the intermediate monodehydroascorbate into ascorbic acid. Taken together, the high ascorbic acid content in *S. androgynus* results from the high expression of related genes in the L-galactose pathway and recycling pathway.

The expansion of *S. androgynus* genes in pathways such as photosynthesis, oxidative phosphorylation, and phenylpropanoid biosynthesis implies the presence of specific physiological and metabolic mechanisms in this plant that are adapted to hot and humid regions (Figure 2D). For instance, the enrichment of the photosynthesis and oxidative phosphorylation pathways implies that *S. androgynus* may possess superior efficiency in the utilization of abundant sunlight and water [44,45]. Additionally, the biosynthesis of phenylpropanoid and benzoxazinoid, typically induced by environmental stimuli [46,47], whose metabolites, such as lignin, have been found to be important in resisting pathogenic invasion and abiotic stress [48], could also confer an advantage for the adaptation of *S. androgynus* to hot and humid environments.

The exceptionally delightful flavor of the leaves is also an attractive feature of *S. androgynus*. Enrichment profiling of the gene family expansions of *S. androgynus* points to its exceptional capacity for biosynthesis of secondary metabolites (Figure 2D). The biosynthetic pathway of phenylpropanoid, one of the main sources of plant color and aroma [49], and terpene compounds, which play an important role in the formation of flavor in species like grape [50] and wintersweet [51], are significantly enriched in *S. androgynus*.

As a medicinal plant, the discovery that the expansion of gene families related to the biosynthesis of secondary metabolites in *S. androgynus* is particularly significant, as the clinically curative effects of medicinal plants are associated with secondary metabolites [52]. Recent studies related to genome assembly in important medicinal plants have significantly advanced our understanding of secondary metabolism. For instance, the genome assembly of the traditional Chinese medicinal plant *Artemisia argyi* [53] has identified genes involved in the biosynthesis pathways of flavonoids and terpenoids, which possess various medicinal properties, including antioxidant, anti-cancer, and anti-inflammatory activities [54,55]. Another example is the genome assembly of *Entada phaseoloides* [56], which has identified genes involved in the biosynthesis of triterpenoid saponins, the main bioactive compounds in *E. phaseoloides*. These whole-genome sequencing studies not only enhance our understanding of the biology and evolution of medicinal plants but also facilitate the discovery of novel drug candidates and the development of sustainable plant-based therapies. As a result, they are crucial for promoting the use of traditional medicinal plants in modern healthcare and for preserving the biodiversity of these valuable genetic resources.

## 4. Materials and Methods

### 4.1. Plant Materials and Sequencing

For genome sequencing, leaves of a single *S. androgynus* were collected from the experimental field of the College of Agriculture, Fujian Agriculture and Forestry University (26°04 N, 119°14 E). High-quality genomic DNA was extracted from the leaves using a modified CTAB method [57].

For Illumina sequencing, a short-read (350 bp) library was constructed and sequenced with the Illumina NovaSeq platform (Illumina, San Diego, CA, USA), and 192.75 Gb of clean reads were obtained. For PacBio sequencing, genomic DNA was fragmented to 15 Kb to construct a long-read library according to the manufacturer’s instructions (Pacific Biosciences, Menlo Park, CA, USA), and then the library was sequenced with the PacBio Sequel II platform. After filtering out the low-quality reads and sequence adapters, we obtained 44.32 Gb of clean subreads with an N50 value of 16.01 kb.

### 4.2. Genome Features Estimation

The genome size of *S. androgynus* was estimated via k-mer and flow cytometry methods. For the k-mer method, the short-reads from the Illumina platform were quality-filtered using fastp [58]. The quality-filtered reads were used for the genome size estimation. We counted the 19 kmers with Jellyfish (v2.2.10) [59] software and calculated the genome characteristics using Genomescope 2.0 [60] software. For the flow cytometry method, nuclei suspensions of *S. androgynus* and *Zea mays* were analyzed using a flow cytometer (BD FACScalibur) and the corresponding software, Modifit (v3.0). The genome size of *S. androgynus* was determined through flow cytometry, with *Z. mays* (~2.3 Gb) serving as the internal standard. The karyotype of *S. androgynus* was determined using the following workflow. Chromosome preparation was performed as described previously [61].

### 4.3. Genome Assembly by CCS Data

A total of 44.32 Gb high-accuracy CCS data were assembled using hifiasm (v0.14) [62] software to obtain the genome sequences, which accounted for ~31.03× of the genome size estimated via k-mer analysis.

### 4.4. Hi-C Technology Help Anchor Contigs

We constructed Hi-C fragment libraries from 300–700 bp insert sizes, as illustrated in Rao et al. [63], and sequencing through Illumina platform. The 192.75 Gb clean Hi-C reads were first truncated at the putative Hi-C junctions and then the resulting trimmed reads were aligned to the assembly results with BWA aligner (v0.7.10). Invalid read pairs, including Dangling-End and Self-cycle, Re-ligation and Dumped products, were filtered using HiC-Pro (v2.8.1) [64]. The unique interaction pairs were used for correction of the contigs onto chromosomes via LACHESIS (https://github.com/shendurelab/LACHESIS (accessed on 22 January 2023)) [65]. The Hi-C data were mapped to these segments using BWA (v0.7.10-r789) [66] software.

### 4.5. Genome Quality Evaluation

The quality of the genome assembly was evaluated using the following three methods. Firstly, the assembled genome was submitted to the Embryophyta database in BUSCO (v5) [67] to evaluate the completeness of the genome. The CEGMA (v2.5) [68] was also used to evaluate the integrity of the final genome assembly. Finally, the short reads from the Illumina platform were aligned to the genome assembly using BWA-MEM (v0.7.10).

### 4.6. Annotation of Repetitive Sequences

The transposon element (TE) and tandem repeats were annotated via the following workflows. The TEs were identified by a combination of homology-based and de novo approaches. We first customized a de novo repeat library of the genome using RepeatModeler (v2.0.1) [69], which can automatically execute two de novo repeat finding programs, including RECON (v1.08) [70] and RepeatScout (v1.0.6) [71]. Then, the full-length long terminal repeat retrotransposons (fl-LTR-RTs) were identified using both LTRharvest (v1.5.9) [72] and LTR_finder (v1.07) [73]. The high-quality intact fl-LTR-RTs and non-redundant LTR library were then produced using LTR_retriever (v2.8) [74]. The flanking sequences on both sides of the LTR were extracted, compared using MAFFT (v7.205) [75] (-localpair -maxiterate 1000), and the distance was calculated via the Kimura model in EMBOSS (v6.6.0) [76]. The insertion time of the LTR elements was estimated according to the formula *T* = *K*/2*r*, where *K* is the divergence rate and *r* is the neutral mutation rate (7 × 10^−9^).

A non-redundant species-specific TE library was constructed by combining the de novo TE sequence library above with the known Repbase (v19.06) [77], REXdb (V3.0) [78] and Dfam (v3.2) [79] databases. The final TE sequences in the *S. androgynus* genome were identified and classified via a homology search against the library using RepeatMasker (v4.10) [80]. The tandem repeats were annotated via the Tandem Repeats Finder (v4.09) and MIcroSAtellite identification tool (MISA v2.1) [81].

### 4.7. Gene Prediction and Annotation

We integrated three approaches, namely, de novo prediction, homology search, and transcript-based assembly, to annotate the protein-coding genes in the genome. The de novo gene models were predicted using two ab initio gene-prediction software tools, Augustus (v2.4) [82] and SNAP (https://github.com/KorfLab/SNAP (accessed on 22 January 2023)) [83], with the training of the best candidate genes obtained via PASA (v2.0.2) software. For the homolog-based approach, GeMoMa (v1.7) [84] software was utilized by using the reference gene model from species including *Arabidopsis thaliana* (TAIR10), *Hevea brasiliensis* (GCA_030052815.1), *Jatropha curcas* (ftp://ftp.kazusa.or.jp/pub/jatropha/ (accessed on 22 January 2023)) and *Manihot esculenta* (https://phytozome.jgi.doe.gov/pz/portal.html#!info?alias=Org_Mesculenta (accessed on 22 January 2023)). For the transcript-based prediction, the RNA-sequencing data in this study were mapped to the reference genome using HISAT2 (v2.0.4) [85] and assembled via StringTie (v1.2.3) [86]. GeneMarkS-T (v5.1) was used to predict genes based on the assembled transcripts. PASA was used to predict genes based on the unigenes that were de novo assembled by Trinity (v2.11) [87]. The gene models from these different approaches were combined using the EVM software (v1.1.1) [88] and updated with the predicted genes from PASA with default parameters. The final gene models were annotated via BLAST (v2.2.31) against the NCBI’s non-redundant protein sequences (NR, 20200921), EggNOG (5.0) [89], TrEMBL (202005) [90], Pfam (33.1) [91], Swiss-Prot (202005) [90], eukaryotic orthologous groups of proteins (KOG, 20110125), gene ontology (GO, 20200615) [92,93] and Kyoto Encyclopedia of Genes and Genomes (KEGG, 20191220) [94] databases. The GO IDs for each gene were obtained from TrEMBL and EggNOG.

The GenBlastA (v1.0.4) [95] program was used to scan the whole genomes after masking the predicted functional genes. The putative pseudogene candidates were then analyzed by searching for non-mature mutations and frame-shift mutations using GeneWise (v2.4.1) [96]. For the non-coding RNA prediction, tRNAscan-SE (v1.3.1) [97] was used to predict tRNA with eukaryote parameters. Identification of the rRNA genes was conducted via Barrnap (v0.9) (https://github.com/tseemann/barrnap (accessed on 22 January 2023)). The miRNA genes were identified using BLAST based on searching the miRBase (release v.21) [98] databases. The snoRNA and snRNA genes were predicted using INFERNAL (v1.1) [99] against the Rfam (release v.12.0) [100] database.

### 4.8. Gene Family Identification

The longest transcript was selected to represent each gene with multiple isoforms. Proteins sequences from *S. androgynus* and 11 other species were used for the family classification using OrthoFinder (v2.4) [101]. The PANTHER (v14) [102] database was used to annotate the obtained gene families.

### 4.9. Phylogenetic Analysis

The protein sequences of the single-copy orthologs were aligned with the MAFFT (v7.205) program (-localpair -maxiterate 1000). Gblocks (v0.91b) [103] (−b5 = h) was used to remove regions with poor sequence alignment or large differences. All the well-aligned gene family sequences of each species were connected end-to-end. IQ-TREE (v1.6.11) [104] were used to construct the phylogenetic tree. ModelFinder [105] was used for the model selection, and the best model was obtained as JTT + F + I + G4, and then the maximum likelihood (ML) method was used to construct the evolutionary tree using this best model.

The MCMCtree with gradient and Hessian parameters from the PAML (v4.9i) [106] package was used to estimate the species divergence time based on the fossil times (*P. trichocarpa* vs. *S. purpurea*, *A. trichopoda* vs. *S. purpurea*, *R. communis* vs. *M. esculenta*, *A. thaliana* vs. *M. esculenta*) from TimeTree [107] (http://www.timetree.org (accessed on 22 January 2023)). *A. trichopoda* was selected as the outgroup and the root of the tree. The ML method, correlated molecular clock, and JC69 model were used to estimate the divergence times. Two repeated calculations were performed to evaluate the consistency. Finally, the phylogenetic tree with divergence times was graphically displayed using MCMCTreeR (v1.1) [108].

### 4.10. Gene Family Expansion and Contraction Analysis

Based on the gene family distribution and the phylogenetic tree with the predicted divergence time of those species, CAFÉ (v4.2) [109] was utilized to analyze the gene family expansion and contraction. In CAFÉ, a random birth and death model is proposed to study the gene gain or loss in gene families across a specified phylogenetic tree. Then, a conditional *p*-value was calculated for each gene family. The criteria for defining significant expansion or contraction of gene families were a family-wide *p*-value < 0.05 and a viterbi *p*-value < 0.05.

### 4.11. WGD Analysis

WGD events were identified via the *Ks* method using WGDI (v0.6.3) [110] software. The time of occurrence of WGD events was calculated using the formula divergence time = *Ks*/2*r* [111], where the *r* (average synonymous substitution rate) of Phyllanthaceae was estimated through the *Ks* distribution of the paralogous genes of *S. androgynus* and *P. cochinchinensis* as 0.4066/(2 × 29.67 × 10^6^) = 6.85 × 10^−9^.

### 4.12. Genome Collinearity Analysis

The gene sequences of the two species were compared using DIAMOND (v0.9.29.130) [112] to identify similar gene pairs (E-value < 1E5, C-score > 0.5, where C-score values were filtered using JCVI (v0.9.13) [113] software). All the genes in the co-linearity blocks were obtained via MCScanX (https://github.com/wyp1125/MCScanX (accessed on 22 January 2023)) [114] (−m5). The macro- and micro-synteny between the *S. androgynus* and *P. cochinchinensis* genomes were visualized using JCVI. The collinearity on the chromosomes of the *S. androgynus* genome was visualized using Circos (v0.69) [115].

### 4.13. Transcriptome Analysis

The transcriptome data concerning the leaf, stem and flower from *S. androgynus* (SRA7983121, SRA7983122, SRA7983124) were downloaded from the NCBI database [116] and used to analyze the expression pattern of genes related to ascorbic acid biosynthesis and the recycling pathways. The adapters were removed and the first 12 low-quality bases of the reads were filtered using Trimmomatic (v0.39) [117]. The genome index was built and the reads were mapped to the genome using HISAT2 (v2.2.1) [118]. Samtools (v1.7) [119] was used to convert the sam file to a bam file. Stringtie (v1.2.3) [86] was applied to calculate the FPKM values, which were used to represent the transcript expression levels.

Ascorbic acid-related resources provided by a previous study [41] was used in this study. Specifically, based on the EggNOG annotation, we anchored the enzyme commission of the protein-coding genes of *S. androgynus* to the genes related to ascorbic acid biosynthesis and the recycling pathways. The expression level of the genes in the related pathways was graphically displayed using the ComplexHeatmap (v2.14.0) package [120] in R (v4.2.2).

## 5. Conclusions

This study presents a chromosome-level *S. androgynus* genome using PacBio sequencing and Hi-C techniques. The *Ks* distribution suggested that *S. androgynus* experienced a recent WGD event about 20.51 Mya and a γ event about 132.95 Mya. Collinearity analysis elucidated a 1:2 relationship between *P. cochinchinensis* and *S. androgynus.* The microsynteny patterns indicated that the expansion of pathways related to phloem development, lignin synthesis, and photosynthesis tended to contribute to the morphological differences among Phyllanthaceae species with various ploidies. This study identified the key regulatory genes of ascorbic acid biosynthesis and the recycling pathways, revealing the mechanism of ascorbic acid accumulation in *S. androgynus*, which provides a genetic basis for its targeted genetic improvement. In addition, this study provided genetic insights into the comprehensive pharmacological activities of *S. androgynus*, encompassing antioxidant activity, geographical distribution patterns and delightful flavor. Taken together, the findings of this study provide a valuable genomic resource for *S. androgynus*, demonstrating the complex ploidy of species within the Phyllanthaceae family. These findings will not only enhance our comprehension of their inherent value but also pave the way for exploitation and utilization.

## Figures and Tables

**Table 1:** Summary statistics for the *S. androgynus* genome assembly and annotation.

Feature	Value
Genome size	1.55 Gb
Contig N50	25.66 Mb
Scaffold N50	58.10 Mb
Anchored to chromosome	1.52 Gb (97.79%)
GC content	33.77%
Number of chromosomes	26
Repetitive sequences	1.15 Gb (77.81%)
Number of protein-coding genes	26,048

## Data Availability

The assembly data were submitted to the Chinese National Genomics Data Center (NGDC; https://ngdc.cncb.ac.cn/ (accessed on 3 September 2023)) under accession number PRJCA018066. The raw data generated in this study were deposited in the National Center for Biotechnology Information (NCBI; https://www.ncbi.nlm.nih.gov/ (accessed on 3 September 2023)) Sequence Read Archive under BioProject accession number PRJNA990470.

## Supplementary Materials

The following supporting information can be downloaded at https://www.mdpi.com/article/10.3390/ijms25010300/s1.
