# Supplementary material for: Polyploid Genome Assembly Provides Insights into Morphological Development and Ascorbic Acid Accumulation of Sauropus androgynus
Source: Int J Mol Sci. 2023 Dec 25;25(1):300. doi: 10.3390/ijms25010300 (PMC10778994; doi:10.3390/ijms25010300)
Supplement: Supplementary file 1 [file ijms-25-00300-s001.zip › Supplementary Figures.pdf]

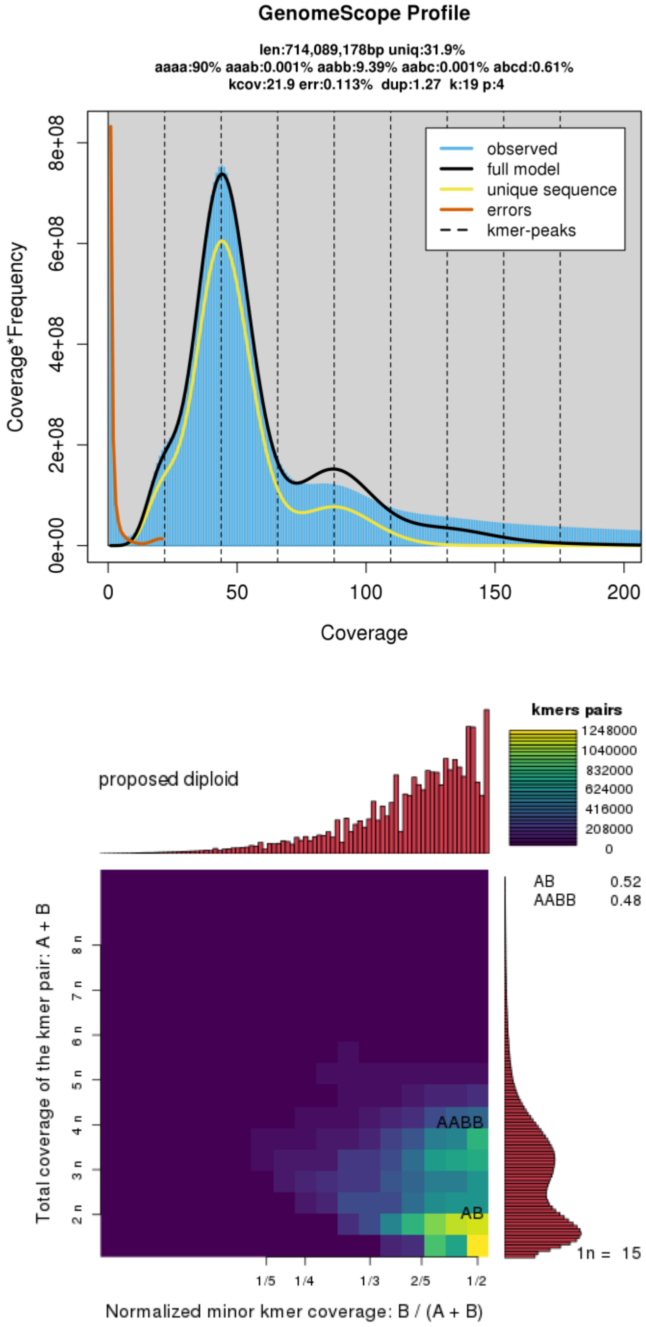

Figure S1. K-mer frequency distribution curve (up) and Smudgeplot (below) of *S. androgynus*.

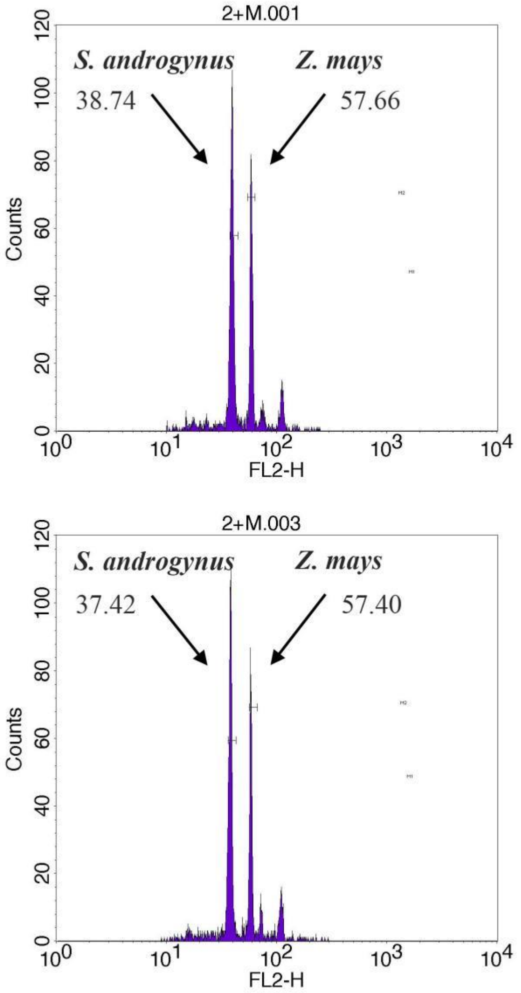

Figure S2. Flow cytometry of *S. androgynus* with *Z. mays* (B73) as reference.

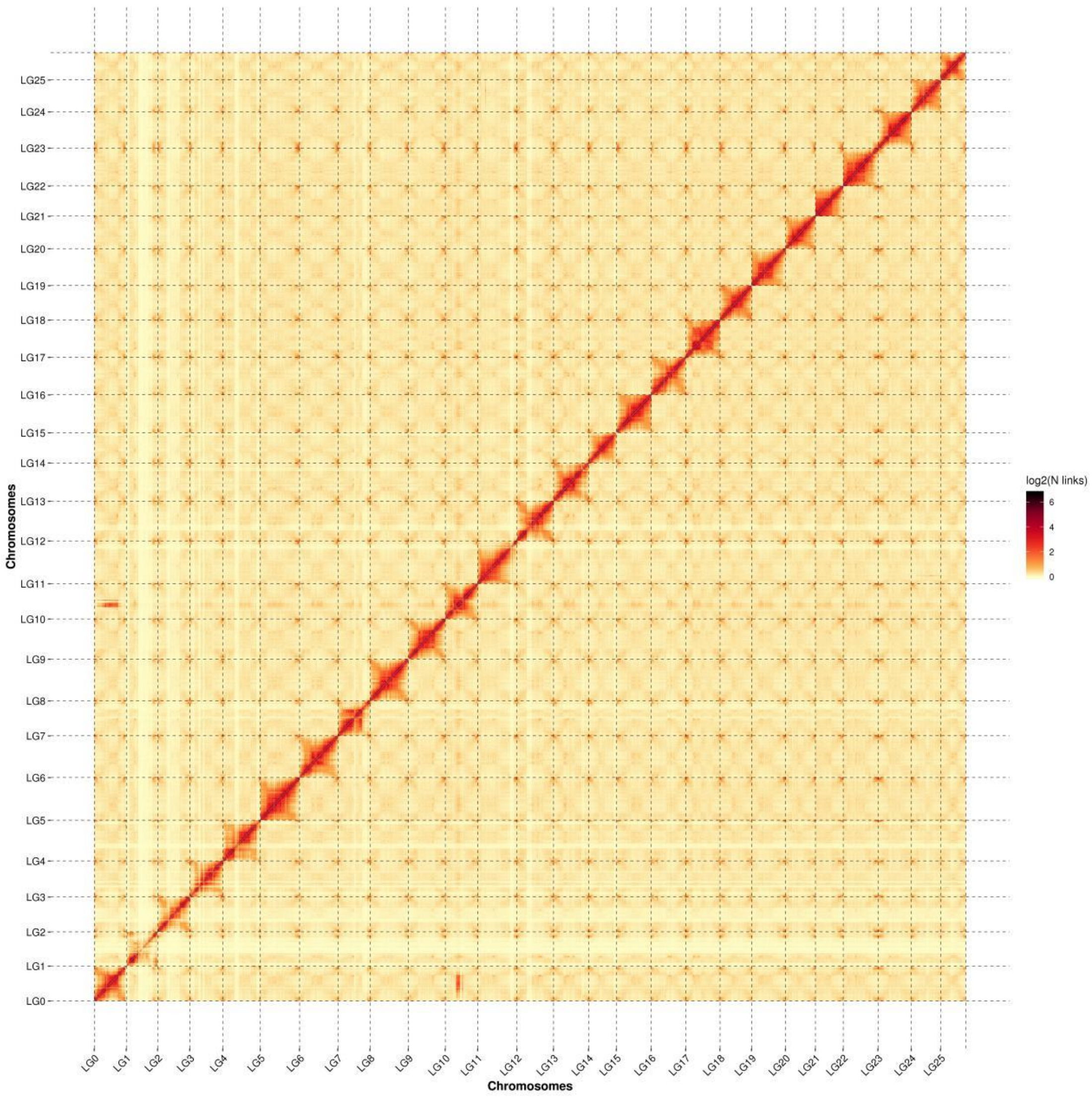

Figure S3. Genome-wide Hi-C heatmap showing chromatin interactions of *S. androgynus* under the resolution of 500 kb.

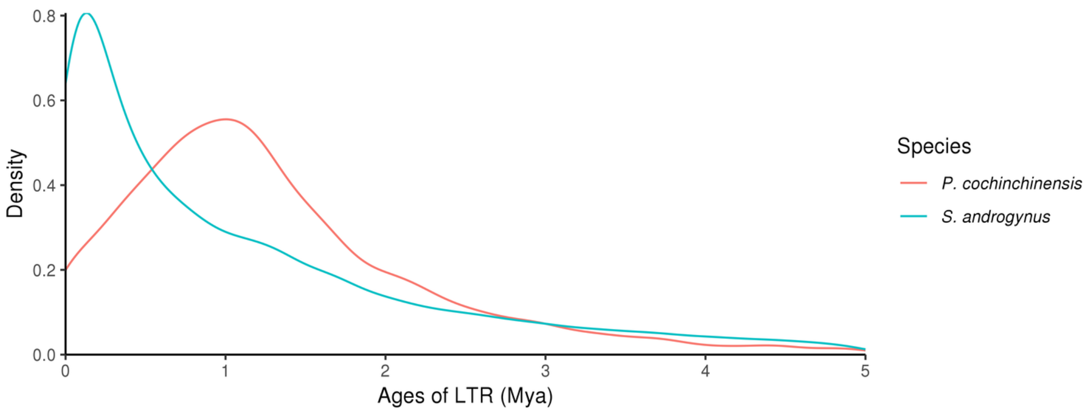

Figure S4. LTR insert time analysis among different species in the Phyllanthaceae family.

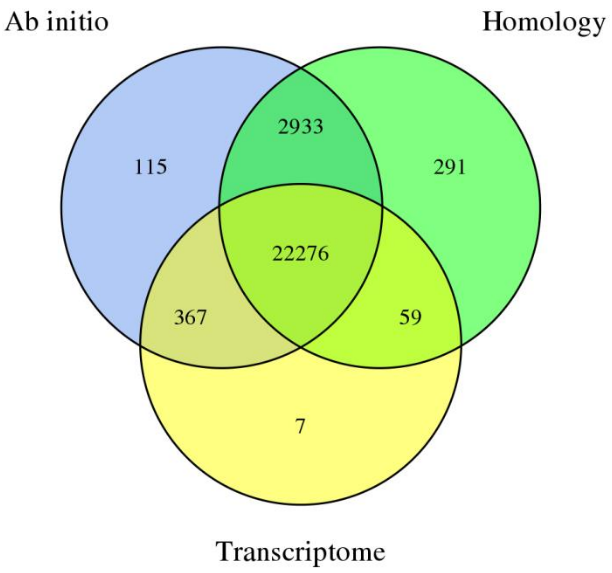

Figure S5. Prediction and annotation of genes in the genome of *S. androgynus*. Number of genes predicted with ab initio, homology, and RNA-seq. All predicted genes were integrated by EVM.

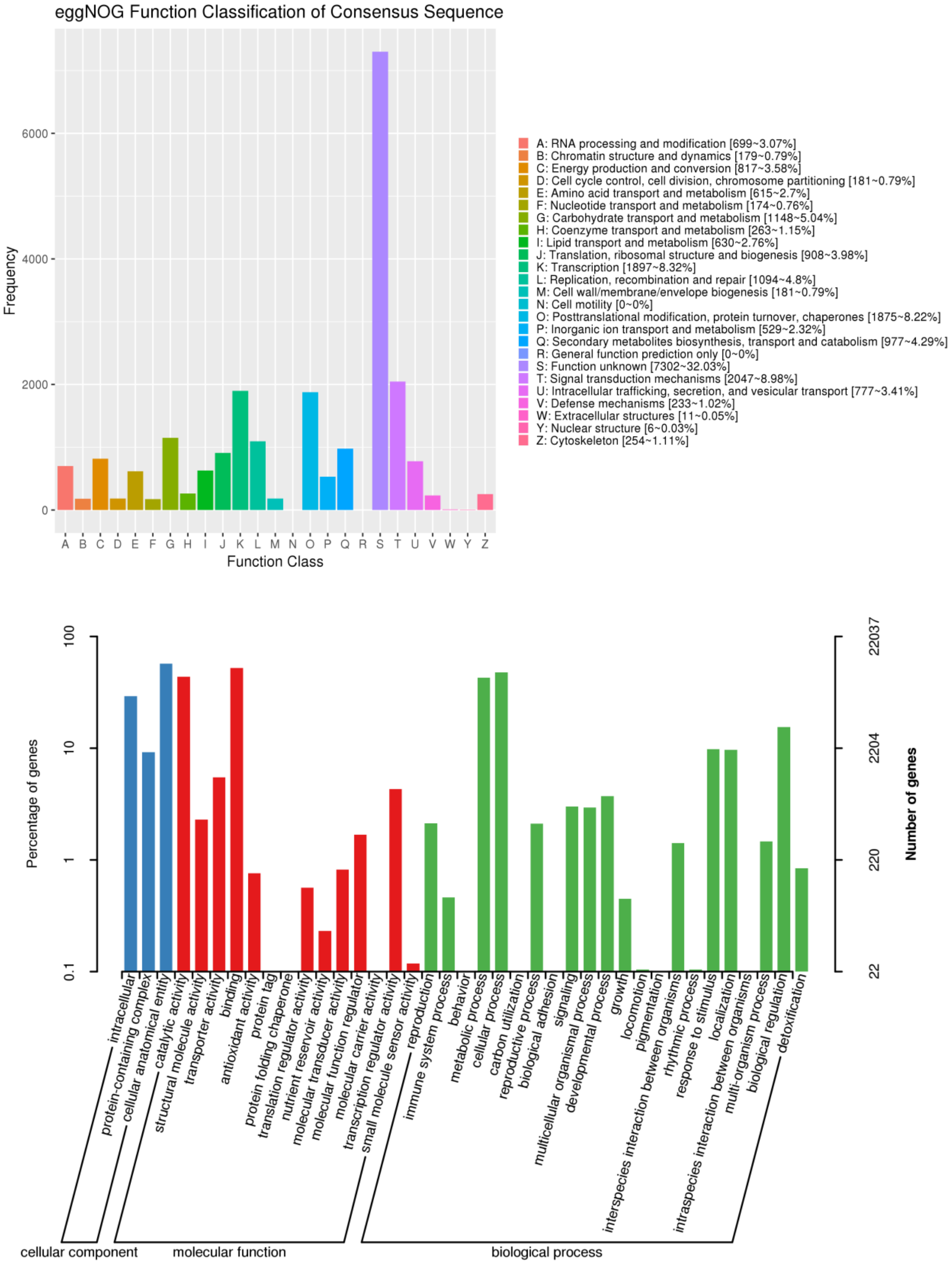

Figure S6. Diagram showing the eggNOG (up) and GO (below) categories of the annotated genes in the genome of *S. androgynus*.

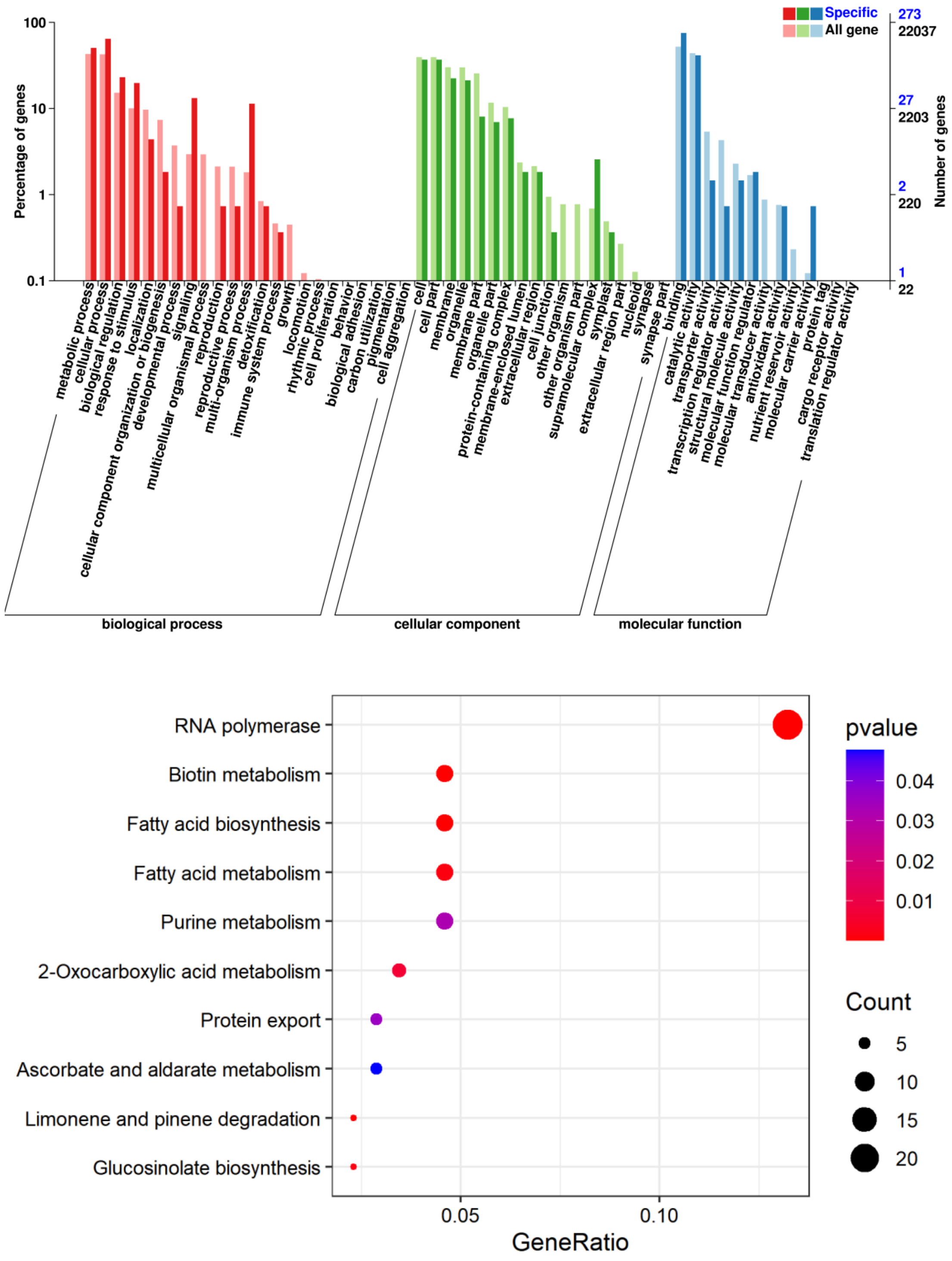

Figure S7. GO (up) and KEGG (below) enrichment of species-specific genes in *S. androgynus*.

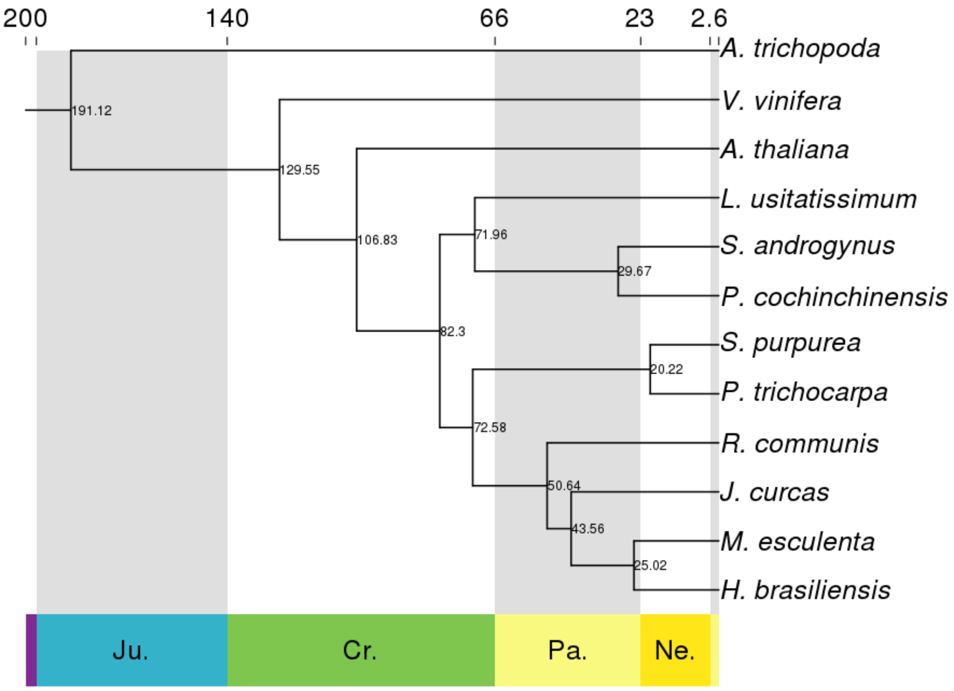

Figure S8. Phylogenetic relationship of 12 species, with divergence times labeled at the top.

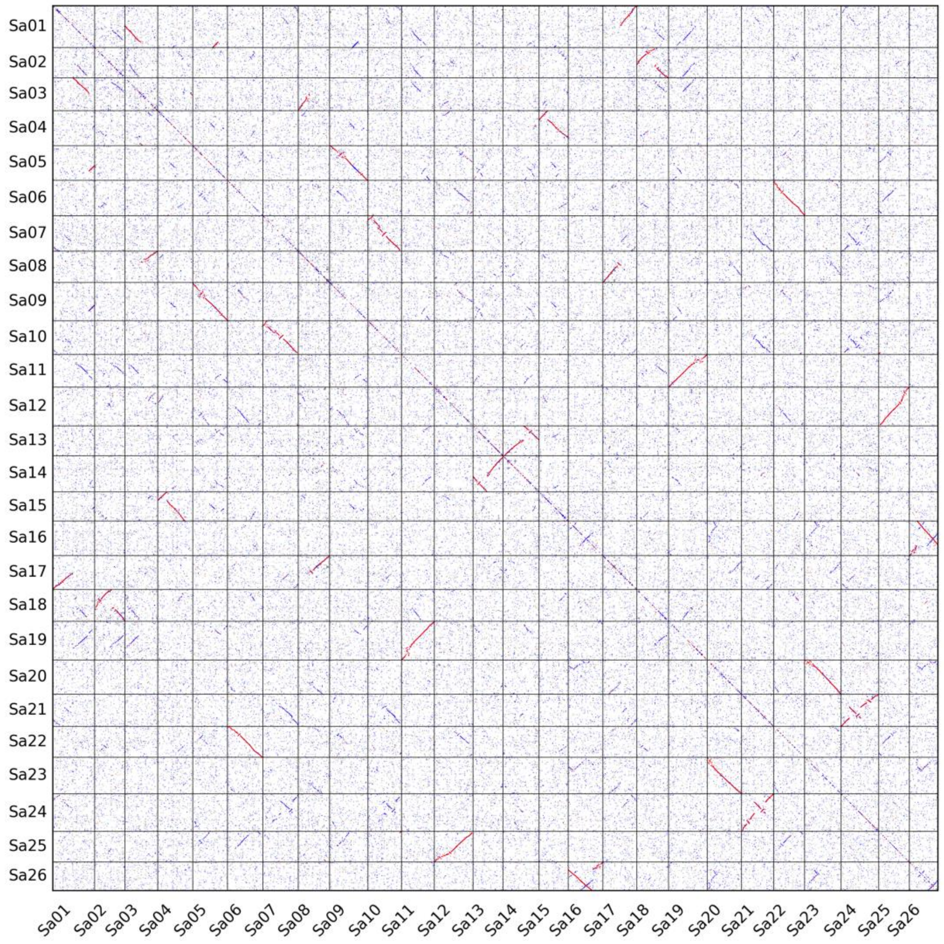

Figure S9. Whole-genome synteny within *S. androgynus* chromosomes.

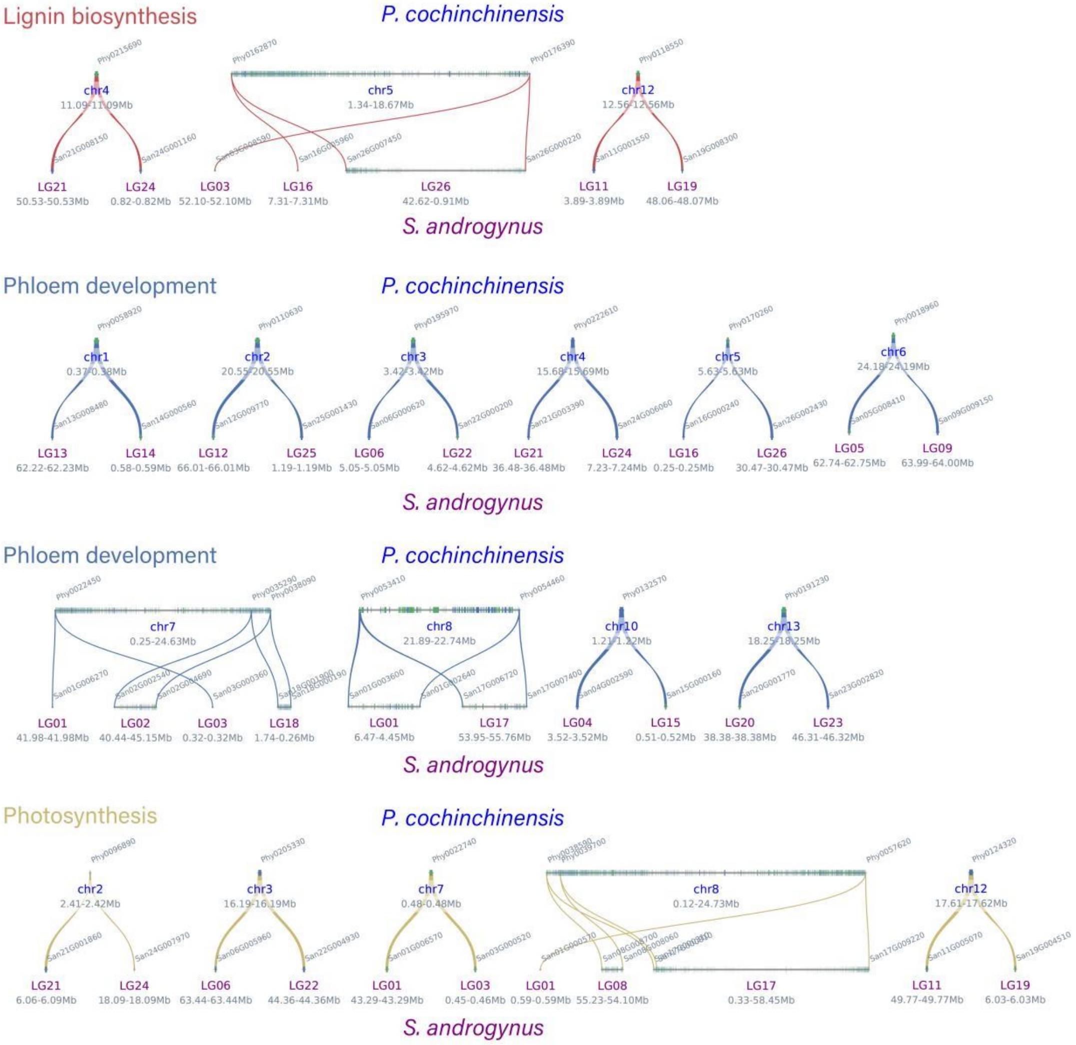

Figure S10. Microsynteny of genes related to morphological development between *S. androgynus* and *P. cochinchinensis*.

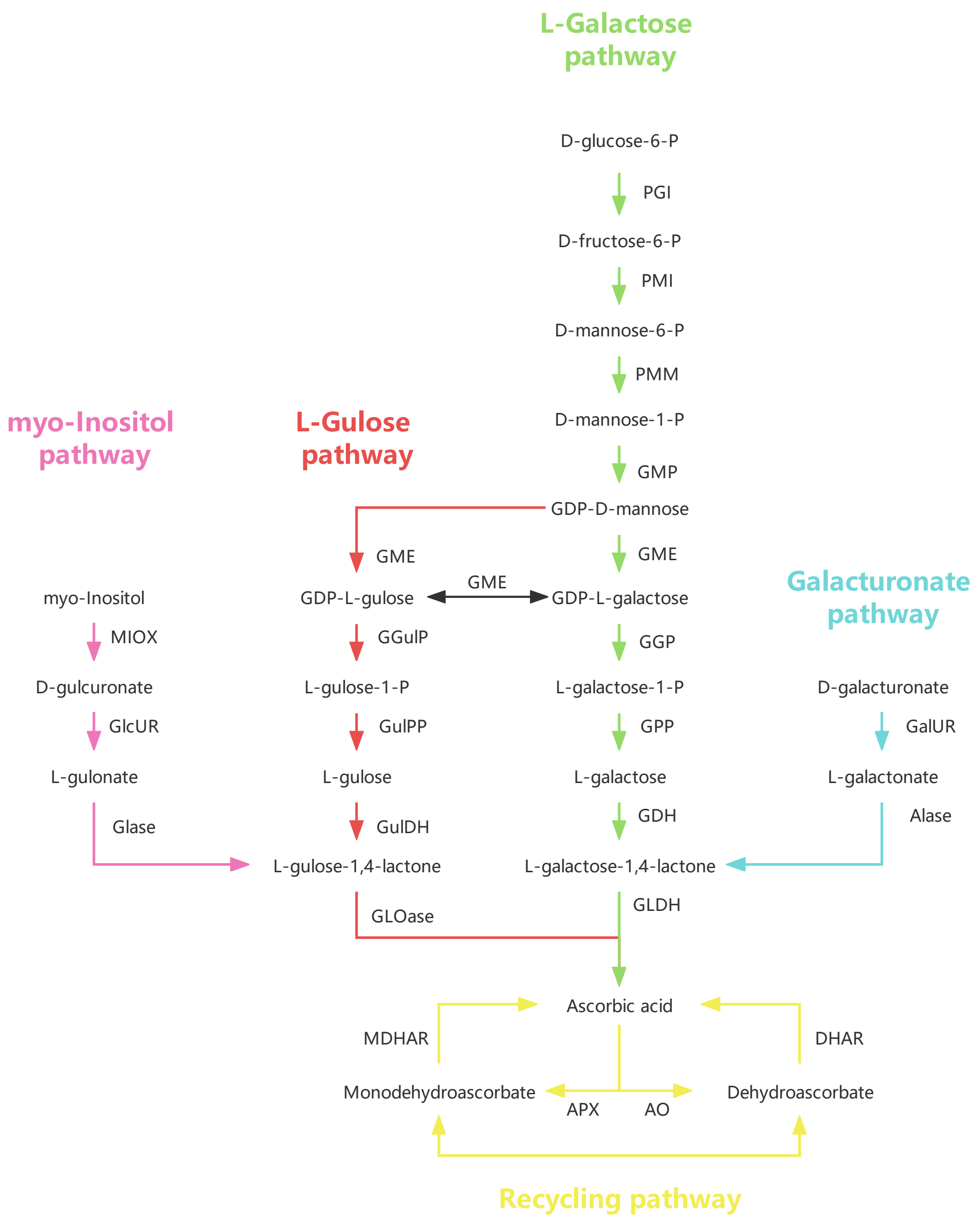

Figure S11. Four biosynthetic pathways and one recycling pathway of ascorbic acid proposed in higher plants. The gene abbreviations with corresponding descriptions are shown in Table S13.
